# The teleNIDA: Early Screening of Autism Spectrum Disorder Through a Novel Telehealth Approach

**DOI:** 10.1007/s10803-023-05927-6

**Published:** 2023-02-23

**Authors:** Valentina Riva, Laura Villa, Francesca Fulceri, Giuseppe Maurizio Arduino, Guido Leonti, Giovanni Valeri, Laura Casula, Leonardo Zoccante, Elena Puttini, Carla Sogos, Mariaelena Presicce, Arianna Bentenuto, Fabio Apicella, Massimo Molteni, Maria Luisa Scattoni

**Affiliations:** 1 Child Psychopathology Unit, Scientific Institute, IRCCS E. Medea, Bosisio Parini, Lecco, Italy; 2 Research Coordination and Support Service, Istituto Superiore di Sanità, Viale Regina Elena 299, 00161 Rome, Italy; 3 Centro Autismo e Sindrome di Asperger ASLCN1, 12084 Mondovì, Italy; 4 Department of Neuroscience, Child Neuropsychiatric Unit, Bambino Gesù Children’s Hospital, IRCCS, Rome, Italy; 5 Child and Adolescent Neuropsychiatry Unit, Maternal-Child Integrated Care Department, Integrated University Hospital of Verona, 37126 Verona, Italy; 6 Child Neuropsychiatry Unit, Department of Human Neuroscience, Sapienza University of Rome, Rome, Italy; 7 Laboratory of Observation, Diagnosis and Education (ODFLab), Department of Psychology and Cognitive Science, University of Trento, 38122 Trento, Italy; 8 Department of Developmental Neuroscience, IRCCS Stella Maris Foundation, Pisa, Italy

**Keywords:** Autism, Telehealth, Early screening, Toddlers

## Abstract

The COVID-19 pandemic has fast-tracked interest in telehealth methods to guarantee the continuity of care of children with Autism Spectrum Disorder (ASD). Store-and-forward telehealth approaches offer the opportunity to facilitate timely screening of ASD, allowing parents to record videos of their child’s behaviors, subsequently shared with clinicians that provide an assessment remotely. This study aimed to examine the psychometric properties of a new telehealth screening tool, the teleNIDA, administered in home settings for remote observation of early signs of ASD in toddlers aged 18–30 months. Results showed good psychometric properties of the teleNIDA, as compared to the gold standard in-person assessment, and the predictive validity on the diagnosis of ASD at 36 months was demonstrated. This study supports the teleNIDA as a promising level 2 screening tool for ASD able to speed up diagnostic and intervention processes.

**Supplementary Information:**

The online version contains supplementary material available at 10.1007/s10803-023-05927-6.

The COVID-19 pandemic has disrupted routines and traditional care practices for children with autism spectrum disorder (ASD) and their families. At the beginning of the coronavirus pandemic, many clinical settings limited their traditional face-to-face assessments or closed entirely. Many in-person visits were suspended, and the restrictions made it challenging to maintain gold-standard evaluations for ASD, which requires face-to-face interactions. This allowed telehealth methods to emerge as a valid alternative for connecting with families with ASD and providing some continuity of care (Narzisi, 2020; Zwaigenbaum & Warren, 2021).

Even before the COVID-19 pandemic, there was growing interest in developing and testing new telehealth methods for ASD to address delays in accessing diagnostic and intervention services (Zwaigenbaum & Warren, 2021). Telehealth can reduce the cost of healthcare, support referral pathways, and facilitate access to services (Gibbs et al., 2021). In addition, telehealth may reduce long waitlists and geographic barriers and help parents be more involved during assessments (Zuckerman et al., 2015).

The onset of social distancing due to the COVID-19 pandemic sparked heightened interest in telehealth methods for screening, diagnosis and/or interventions with children with ASD. Previous studies have mainly focused on the effectiveness of using telehealth technologies in intervention practices. Limited research has been conducted in the literature regarding the use of telehealth for screening and diagnosis of ASD (Lindgren et al., 2020; Sutherland et al., 2018; Wacker et al., 2013). Overall, a recent review (Stavropoulos et al., 2022) showed that telehealth methods can be accurate compared to in-person assessments and have acceptable sensitivity and specificity values.

Telehealth approaches include synchronous and asynchronous methods. In synchronous approaches (Real-Time methods), such as “live” videoconferencing, clinicians guide the child’s caregiver in a series of activities and observe elicited behaviors in real-time.

One of the most used methods is the TELE-ASD-PEDS (Corona et al., 2021a, 2021b; Wagner et al., 2021, 2022), which was designed for clinicians with expertise in early diagnosis who guide parent–child interactions using familiar toys and materials. This tool was developed to augment diagnostic decision-making by expert clinicians who administer comprehensive ASD evaluations. Asynchronous telehealth methods (Store-and-Forward methods) rely on uploading videos of child-caregiver interactions to web portals, where the records are stored and subsequently shared with clinicians. Previous studies have demonstrated parents’ ability to record videos of their children’s behavior in home settings and share them with clinicians (Nazneen et al., 2015). Compared to the real-time telehealth approach, the asynchronous methods may minimize the need to coordinate schedules with clinicians and parents can record videos over a day or multiple days, based on their convenience.

In the ASD context, various asynchronous instruments have been tested to conduct in-home observations of children in the first few years of their lives. Some instruments are designed for screening or level 2 screening, whereas others are designed for diagnostic purposes. The most common instruments in this context are the Naturalistic Observation Diagnostic Assessment (NODA; Nazneen et al., 2015; Smith et al., 2017), the Systematic Observation of Red Flags (SORF; Dow et al., 2017), and the Brief Observation of Symptoms of Autism (BOSA; Dow et al., 2021).

The NODA consists of in-home videos of different everyday scenarios (i.e., family mealtime, playtime with others, playtime alone, parent concerns) according to specific instructions. Parents upload the videos to the NODA web platform and clinicians code the child’s behaviors according to a DSM-5 checklist (i.e., ASD or not ASD; Carpenter, 2013). This instrument was used for diagnostic purposes in a sample of 51 children (40 children assessed for ASD suspicion and 11 typically developing children) aged 18 months to 6 years old. The results showed a significant agreement between NODA scores and in-person evaluations for diagnostic categories (ASD, non-ASD) based on DSM-5 criteria (APA, 2013), with a sensitivity of 84.9% and a specificity of 94% (Smith et al., 2017).

The SORF offers screenings during home-observations of toddlers with ASD and developmental delay (Dow et al., 2020) or infants potentially at risk of ASD (Pileggi et al., 2021). This consists of in-home videos recorded during at least five different everyday activities (i.e., play with toys, play with people, meals and snacks, caregiving, and family chores) for at least 30 minutes. This instrument has been used for screening purposes and to assist in diagnostic decision-making (Dow et al., 2020; Pileggi et al., 2021). The procedures and scoring are designed to detect 22 red flags for autism based on DSM-5 diagnostic criteria (Dow et al., 2020). The study examined the psychometric properties of the SORF in a sample of 228 toddlers (84 with ASD, 82 with developmental delay, and 62 with typical development) aged 18 to 24 months. The results based on diagnostic classifications (ASD vs. nonspectrum) revealed a specificity of 63% and sensitivity of 73% for items related to the social communication/interaction domain and a specificity of 54% and sensitivity of 70% for items related to restricted/repetitive behaviors. Furthermore, six items obtained the best psychometric properties (i.e., poor eye gaze directed to faces, limited showing and pointing, limited coordination of nonverbal communication, less interest in people than objects, repetitive use of objects, and excessive interest in particular objects, actions, or activities) and showed higher sensitivity (77%) and specificity (72%) scores (Dow et al., 2020). The provisional cut-off scores for total and subdomains supported the clinical utility of this instrument.

Finally, the BOSA is designed as a synchronous and asynchronous method and consists of live or video-recorded parent–child interaction for about 12–14 min using standardized materials within an Autism Diagnostic Observation Schedule—Second Edition (ADOS-2) coding framework. This instrument provides a naturalistic social context using materials chosen according to the individual’s age, language, and developmental level. The scoring could be used to determine the initial risk for ASD as a level 2 screening or in conjunction with other diagnosing methods (Dow et al., 2021).

When the pandemic halted clinical services in Italy, the clinical staff within the Network for Early Detection of Autism Spectrum Disorders (NIDA network) reviewed available literature to identify a feasible and reliable instrument for clinical and research purposes. The NIDA network is the largest multi-center and multi-disciplinary network in Italy and aims to guide observational studies and surveillance programs for early screening of ASD (for details, see Caruso et al., 2021; Costanzo et al., 2015; Micai et al., 2020; Riva et al., 2021). The NIDA staff reported the need for a reliable measure that performs as well as the gold standard in diagnosing autism, even in a shorter timeframe than other asynchronous tools, and in different everyday naturalistic settings. Indeed, validity parameters on the SORF are based on one-hour home observation and validation data from a shorter version is not available. Furthermore, the BOSA is based on ADOS-2 standardized activities/materials and does not consider observations in different everyday settings. Home observation tools during everyday activities may offer the opportunity to integrate additional information on the child's behavior.

Based on these needs and the literature review of validated screening programs, the NIDA staff developed a new store-and-forward telehealth instrument, called teleNIDA, and tested it as a level 2 screening in a sample of children at risk of ASD either because they are already under observation for developmental concerns, or because they are at elevated likelihood of developing ASD (i.e., siblings of children with ASD). The teleNIDA is designed for toddlers aged 18 to 30 months of age and provides home-setting observations of potential atypical behaviors associated with ASD. It guides parents to capture 5-min videos of their child during four everyday activities (i.e., free-play, play with parents/caregivers, mealtime, and book sharing) which allows clinicians to observe children in familiar and naturalistic environments. Since previous studies emphasized the relevance of providing specific instructions for parents in telehealth assessments (Nazneen et al., 2015), we elaborated simplified written instructions for parents accompanied by clear images. To code atypical behaviors, clinicians completed a rating form based on and adapted from the 22-item SORF scoring (Dow et al., 2020).

This study is the first to assess the psychometric properties of the teleNIDA. The convergent validity of the teleNIDA and ADOS-2 and the inter-rater reliability values were computed for a sample of Italian toddlers aged 18 to 30 months. The validity parameters and scores for the teleNIDA domains and items (i.e., sensitivity, specificity, positive and negative predictive values) were reported and the optimal cut-offs were provided for clinical utility and practices. Finally, we also examined the predictive validity of the teleNIDA on the clinical best estimate diagnosis at 36 months (ASD vs. nonspectrum) to help clinicians in the referral process for a comprehensive diagnostic evaluation.

## Methods

### Participants

The sample consists of 51 participants ranging from 18 to 30 months (mean = 23.37 months; SD = 3.80; Males = 32; Females = 19), including 30 siblings of children with ASD (mean = 23.17 months; SD = 2.88; Males = 19; Females = 11) who enrolled in the longitudinal surveillance of NIDA network and 21 toddlers referred for suspicion of ASD (mean = 24.90 months; SD = 4.11; Males = 13; Females = 8). No sex and age differences were found between the two groups (sex: *χ*^2^(51) = 0.01; *p* = 0.917; age: F(50) = 3.164; p = 0.081). All the children were scheduled for telehealth and in-person evaluations at the clinical NIDA center from September 2020 to July 2021. Participants were recruited from five Italian pediatric institutes within the NIDA network: the Institute for Research, Hospitalization and Health Care (acronym in Italian IRCCS) Medea in Lecco (n = 19), the IRCCS Bambino Gesù in Rome (n = 10), Polyclinic Umberto I Hospital in Rome (n = 5), Maternal-Child Integrated Care Department, Integrated University Hospital of Verona (n = 5), Centro Autismo e Sindrome di Asperger in Mondovì (n = 12).

Each participant underwent a comprehensive clinical evaluation, including the teleNIDA, the ADOS-2, and the Griffiths Mental Development Scales-Extended Revised (GMDS-ER). All tests were administered by trained clinicians and researchers with extensive experience using gold standard diagnostic tools.

The Ethical Committee of the Istituto Superiore di Sanità (Rome, Italy) approved all parts of the experimental protocol and methods described in this paper. Informed consent was obtained from all participants prior to inclusion. The study was conducted in accordance with ethical standards (Declaration of Helsinki).

### Materials

#### The teleNIDA

The teleNIDA is a store-and-forward instrument developed for remote home-setting observation of children aged 18 to 30 months who have been referred due to concerns for ASD. Parents/caregivers were asked to make 5-min video recordings of four everyday activities (i.e., free play, play with parents/caregivers, mealtime, and book sharing) for a total of about 20-min of recording. Parents were provided with ad hoc written instructions accompanied by clear images to guide them in interaction and recording procedures (see Online Resource 1 “teleNIDA guidelines for parents/caregivers”). If needed, the clinicians could send additional verbal information and recording instructions about the environment setup to help parents.

#### Scoring and Interpretation

Once the videos were collected, clinicians completed the teleNIDA rating form, which was modified and adapted from the SORF (Dow et al., 2020). The teleNIDA scoring procedures were shared within the NIDA network and the rating forms were coded by clinicians with extensive experience in diagnosing ASD. No specific training on teleNIDA is required.

The rating form consists of 22 observational items (11 items from the social communication domain and 11 items from the restricted/repetitive behaviors domain) that were coded using a 4-point Likert scale (from 0 to 3). For items describing atypical behavior, a code of “0” indicates typical behavior, a code of “1” indicates atypical behavior at the subclinical level, a code of “2” indicates moderate atypical behavior (one/two times during the activity), and a code of “3” indicates robust atypical behavior (more than two times during the activity). Conversely, for items describing the lack of typical behaviors, a code of “2” indicates rare typical behavior and a code of “3” indicates the absence of typical behavior (e.g., many/several/few/no instances of sharing reciprocal social play). A code of “8” (i.e., not codable) may be used for three items (items 4, 8, and 14) when behavior cannot be assessed because of insufficient evidence (e.g., repetitive intonation and use of consonants cannot be assessed if the child does not vocalize, and response to their name when called cannot be assessed if name is not called at least two times). The teleNIDA scoring procedure requires that some items (n = 8; i.e., 1, 2, 4, 5, 6, 7, 11, 18) are not coded in the activities when there is both a low opportunity to observe the behavior and a high risk of bias (e.g., eye contact is coded during play with parents/caregivers but is not coded during the video recording of book sharing because the child is often on the parent’s lap). The other items (n = 14) were coded in all 4 activities. For each item level, a final average score was provided (see Online Resource 1 “teleNIDA scoring”). The items removed from specific activities were determined by an initial analysis of a sample of 10 toddlers; at least 75% received a score of N/A or “8” (i.e., not codable).

#### Inter-Rater Reliability of the teleNIDA

Inter-Rater Reliability and Intra-Class Correlations (ICC) were computed using a sample of 40 videos of ten participants aged from 18 to 30 months. Video recordings were coded by fourteen experienced clinicians from seven NIDA centers who were blind to the diagnostic status. The ICC of the teleNIDA was excellent for the total scores (ICC = 0.960) and the two subdomains (communication and social interaction ICC = 0.964; restricted/repetitive behaviors ICC = 0.886).

#### Autism Diagnostic Observation Schedule—Second Edition (ADOS-2)

The ADOS-2 is a semi-structured assessment of communication, social interaction, and restricted/repetitive behaviors for individuals suspected of having ASD (Lord et al., 2012). It includes five modules depending on developmental, age, and language levels.

In this study, we used the ADOS Toddler module and ADOS-2 Module 1 to evaluate the severity of ASD symptoms. The Calibrated Severity Score (CSS) was calculated for each participant using the total, Social Affect (SA) and Restricted Repetitive Behaviors (RRB) scores. The CSS ranges from 1–10 and makes it possible to compare different modules of ADOS-2 by controlling for participants’ age and language levels (Esler et al., 2015; Gotham et al., 2009). The examiners who administered the ADOS-2 were not the same ones who directly scored the teleNIDA. The clinicians who scored the teleNIDA were blind to the results of the ADOS-2 and vice-versa.

#### Griffiths Mental Development Scales-Extended Revised (GMDS-ER)

The GMDS-ER is a developmental assessment procedure that includes five subscales (i.e., Locomotor, Personal-Social, Hearing and Language, and Eye and Hand Coordination and Performance) and a developmental quotient. In this study, general developmental quotients were provided (Griffiths, 1996). Table 1 shows descriptive statistics of the clinical profiles.

**Table 1:** Participant clinical characteristics (n = 51)

	Total sample mean (SD)
GMDS-ER Developmental Quotient	86.49 (19.73)
ADOS-2 CSS	
Social affect	5.22 (2.53)
Restricted/repetitive behaviors	4.39 (2.76)
Total	4.78 (2.77)

*GMDS-ER* Griffiths Mental Development Scales, Extended Revised, *CSS* Calibrated severity scores

### Statistical Analysis

Pearson correlations were computed for the teleNIDA and the ADOS-2 to analyze the degree of association between the measures. The internal consistency of the items in each of the two areas of the teleNIDA (i.e., Social Communication and Interaction, Restricted and Repetitive Behaviors) was calculated and Cronbach’s alpha coefficients were determined.

Receiver Operating Characteristic (ROC) curves compare sensitivity versus specificity across a range of values to predict a binary outcome. Using the ADOS-2 scores as the dichotomous target variable (1: total ADOS-2 CSS ≥ 6 points; 0: otherwise) and the teleNIDA as the independent variable, we carried out ROC curve analyses with SPSS software. ROC curves were created for the subdomains (Social Communication and Interaction, Restricted and Repetitive Behaviors) and the total scores to examine how well the teleNIDA differentiates children at risk for ASD compared to children at low risk for ASD (as measured by ADOS-2).

A ROC curve analysis was conducted to provide specificity, sensitivity, and optimal clinical cut-off scores for the teleNIDA. Area Under the Curve (AUC) scores were provided to show the strength of discrimination between children with high-level vs low-level concerns. An AUC of 0.50 means that the classifier (the teleNIDA) cannot distinguish between high-risk and low-risk classes, whereas an AUC of 1 means that the classifier (the teleNIDA) is able to perfectly distinguish between classes. Recommended and optimal cut-off scores were selected to prioritize sensitivity and maintain adequate specificity level. Positive Predictive Values (PPVs) and Negative Predictive Values (NPVs) were calculated using the optimal cut-offs. Furthermore, AUC values were provided at the item level to examine how well each item could discriminate between toddlers with high-level vs. low-level concern classifications.

Finally, we examined the predictive validity of the teleNIDA on the clinical best estimate diagnosis at 36 months. To determine how well the teleNIDA discriminated ASD versus nonspectrum groups, we therefore estimated AUC, sensitivity, specificity, PPVs, and NPVs.

## Results

First, we examined the correlations between the teleNIDA (Social Communication and Interaction, Restricted/Repetitive Behaviors, total scores) and the ADOS-2 Calibrated Severity Scores (SA CSS, RRB CSS, and total ADOS-2 CSS). After applying Bonferroni correction to multiple comparisons (0.05/9 = 0.006), we found significant correlations among all measures (Table 2). Large positive associations emerged between the teleNIDA Social Communication and Interaction and the ADOS-2 SA CSS (r(51) = 0.783; p < 0.001), the teleNIDA Restricted/Repetitive Behaviors and the ADOS-2 RRB CSS (r(51) = 0.465; p < 0.001), and the total teleNIDA and the ADOS-2 CSS (r(51) = 0.817; p < 0.001). These results provided evidence of good convergent validity between the two measures.

**Table 2:** Correlations between teleNIDA and ADOS-2 scores

	ADOS-2 CSS social affect	ADOS-2 CSS restricted and repetitive behaviors	ADOS-2 CSS total
teleNIDA social communication and interaction	0.783**	0.541**	0.780**
teleNIDA restricted/repetitive behaviors	0.586**	0.465**	0.597**
teleNIDA total	0.802**	0.620**	0.814**

*CSS* calibrated severity scores

**p < .006 (0.05/9; Bonferroni correction threshold)

### Convergent Validity

**Fig. 1:**
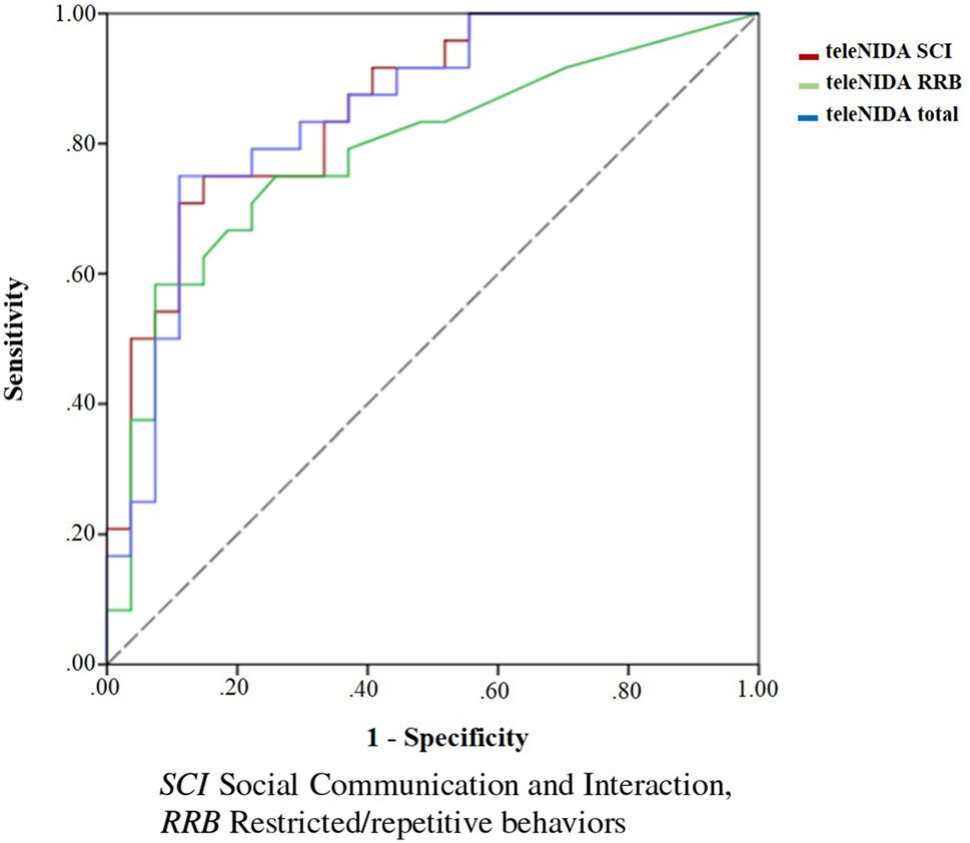
The ROC curves of the teleNIDA

To establish the optimal cut-offs for the teleNIDA, we used ROC-based methods to calculate the sensitivity and specificity of different cut-offs. Table 3 shows the ROC results, which demonstrate good validity scores for the teleNIDA (indicated by an AUC > 0.80; see Glascoe, 2005), and Fig. 1 shows the ROC curves of the teleNIDA scores.

**Table 3:** Validity parameters of the teleNIDA

	AUC [CI 95%]	Optimal cut-off	Sensitivity	Specificity	PPV	NPV
teleNIDA social communication and interaction	0.86 [0.76, 0.96]	13	0.83	0.67	0.63	0.88
teleNIDA restricted/repetitive behaviors	0.79 [0.66, 0.92]	2	0.75	0.74	0.74	0.75
teleNIDA total	0.85 [0.74, 0.96]	15	0.83	0.70	0.68	0.83

*AUC* area under curve, *CI* confidence interval, *PPV* positive predictive value, *NPV* negative predictive value

In addition, we computed the PPV, the probability that children who screened positive on the teleNIDA obtained moderate-to-severe ADOS-2 concern classification, and the NPV, the probability that children who screened negative on the teleNIDA obtained low-to-moderate ADOS-2 concern classification. The PPV was 63% for social communication/interaction, 74% for restricted/repetitive behavior and 68% for total teleNIDA scores. The NPV was 88% for social communication/interaction, 75% for restricted/repetitive behaviors, and 83% for total teleNIDA scores (see Table 3). Furthermore, Cronbach’s alpha coefficients were computed for the two teleNIDA subscales. We found good levels of internal consistency for Social Communication and Interaction (α = 0.942) and acceptable levels for Restricted/Repetitive Behaviors (α = 0.773).

**Fig. 2:**
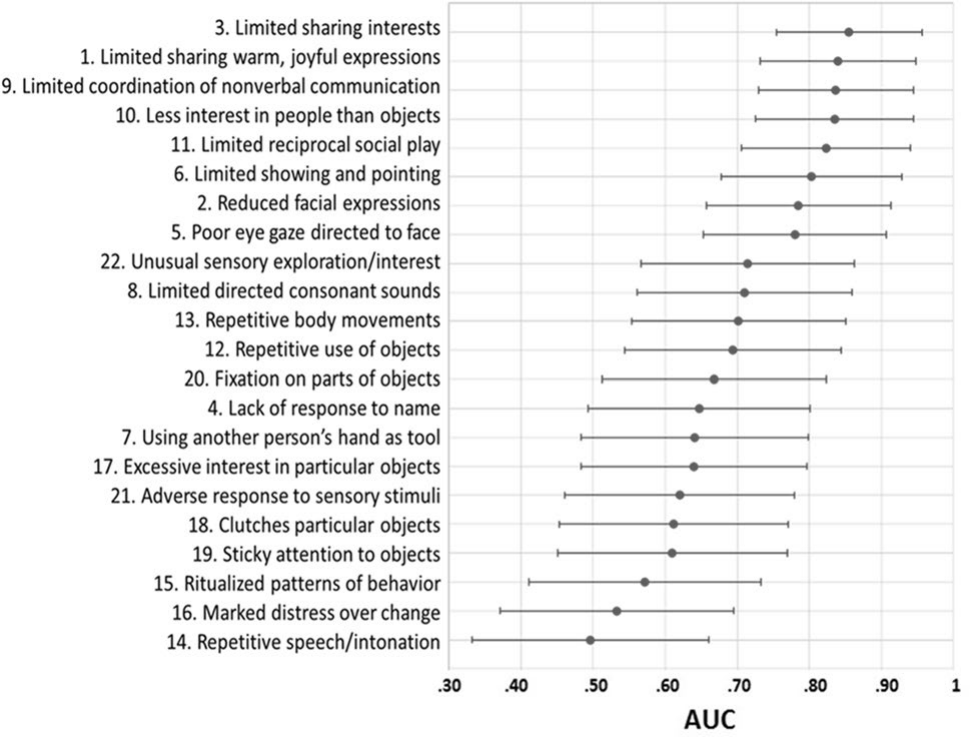
Graphical representation of the AUC values at item level. Items are arranged in descending order, from the greater to the lesser AUC value

The ROC curves were calculated at the item level to examine each item’s strength when differentiating between toddlers with moderate-to-severe levels (or autism; n = 24), based on the ADOS-2, compared to toddlers with little-to-no concern/mild-to-moderate classes in ADOS Toddler Module (or spectrum/non spectrum in ADOS-2 Module 1; n = 27). The results showed that 13 items had significant AUC values (9 within the social communication/interaction domain and 4 within the restricted/repetitive behaviors domain) with AUC greater than 0.65; 8 of them (within the social communication and interaction domain) were the best performing items with AUC values greater than 0.75. This demonstrated limited sharing of warm/joyful expressions, reduced facial expressions, limited sharing of interests, poor eye contact directed at faces, limited showing and pointing, limited coordination of nonverbal communication, less interest in people than objects, and limited reciprocal social play (see Table 4; Fig. 2 for details).

**Table 4:** Item level AUC values

	AUC	SE	p-value	[CI 95%]
1. Limited sharing warm, joyful expressions	0.840**	0.055	< .001	[0.731, 0.948]
2. Reduced facial expressions	0.785**	0.065	0.001	[0.657, 0.913]
3. Limited sharing interests	0.855**	0.051	0.000	[0.754, 0.956]
4. Lack of response to name	0.647	0.079	0.077	[0.492, 0.801]
5. Poor eye gaze directed to face	0.780**	0.065	0.001	[0.653, 0.907]
6. Limited showing and pointing	0.803**	0.064	< .001	[0.678, 0.928]
7. Using another person’s hand as tool	0.641	0.080	0.089	[0.483, 0.799]
8. Limited directed consonant sounds	0.710*	0.076	0.011	[0.561, 0.859]
9. Limited coordination of nonverbal communication	0.837**	0.055	< .001	[0.729, 0.944]
10. Less interest in people than objects	0.835**	0.056	< .001	[0.725, 0.945]
11. Limited reciprocal social play	0.823**	0.060	< .001	[0.706, 0.940]
12. Repetitive use of objects	0.694*	0.077	0.019	[0.544, 0.844]
13, Repetitive body movements	0.702*	0.076	0.015	[0.553, 0.851]
14. Repetitive speech/intonation	0.496	0.084	0.911	[0.332, 0.660]
15. Ritualized patterns of behavior	0.572	0.083	0.386	[0.410, 0.733]
16. Marked distress over change	0.533	0.083	0.690	[0.371, 0.695]
17. Excessive interest in particular objects	0.640	0.080	0.090	[0.483, 0.797]
18. Clutches particular objects	0.612	0.081	0.176	[0.452, 0.771]
19. Sticky attention to objects	0.610	0.081	0.186	[0.450, 0.769]
20. Fixation on parts of objects	0.668*	0.079	0.042	[0.513, 0.824]
21. Adverse response to sensory stimuli	0.620	0.081	0.147	[0.461, 0.779]
22. Unusual sensory exploration/interest	0.714*	0.075	0.010	[0.566, 0.862]

*AUC* area under curve, *SE* standard error, *CI* confidence interval

*p < .05; **p < .01

### Predictive Validity

To improve the clinical utility of the teleNIDA, we used ROC-based methods to determine how well the teleNIDA scores discriminate between children with ASD and non-autistic children at 36 months. Toddlers were classified as having ASD if they had a 36-month clinical best estimate diagnosis of autism according to the Diagnostic and Statistical Manual of Mental Disorders, Fifth Edition (DSM-5; APA 2013). Based on the clinical best estimate diagnosis, the sample consisted of 21 autistic (17 males) and 30 non-autistic children (15 males). Among non-autistic children, 6 (two males) showed atypical development, including language and developmental delay. More specifically, the sample of siblings at elevated likelihood of being autistic included 20% of children with a diagnosis of ASD (n = 6), whereas the sample of toddlers referred for suspicion of ASD included 71% of children with a diagnosis of ASD (n = 15).

The results revealed that the teleNIDA total scores provided good discrimination (see Table 5) between the ASD and non-autistic groups (i.e., atypical development combined with typical groups), with AUC values of 0.81, with a sensitivity of 0.76, a specificity of 0.70, PPV of 0.63, and NPV of 0.78. The Social Communication and Interaction subscale demonstrated good discrimination (AUC = 0.81), yielding a sensitivity of 0.71, specificity of 0.70, PPV of 0.71, and NPV of 0.78. Finally, the Restricted/Repetitive Behaviors subscale demonstrated AUC = 0.77, with a sensitivity of 0.71, a specificity of 0.80, PPV of 0.65, and NPV of 0.79.

**Table 5:** ROC curve analysis comparing children with ASD (n = 21) versus nonspectrum children (n = 30)

	AUC [CI 95%]	Optimal cut-off	Sensitivity	Specificity	PPV	NPV
teleNIDA social communication/interaction	0.81 [0.68, 0.94]	15	0.71	0.70	0.71	0.78
teleNIDA restricted/repetitive behaviors	0.77 [0.64, 0.91]	2	0.71	0.80	0.65	0.79
teleNIDA total	0.81 [0.68, 0.94]	17	0.76	0.70	0.63	0.78

*ROC* receiver operating characteristics, *AUC* area under curve, *CI* confidence interval, *PPV* positive predictive value, *NPV* negative predictive value

## Discussion

The present study aimed to examine the psychometric properties of the teleNIDA, a new store-and-forward telehealth method tested for level 2 screening of ASD. It guides parents to collect videos of their child’s behavior and subsequently share them with clinicians to provide an assessment of early ASD signs. We developed a new ad hoc protocol to direct parents in the videotaping process, ensuring that videos are comparable among participants and evoke significant behaviors to support clinical judgment. Notably, our results related to inter-rater reliability indicated 89–96% accuracy among 14 raters from seven different pediatric institutes in five regions in Italy. This strong agreement among raters with expertise in ASD may ensure the feasibility, repeatability, and reproducibility of the teleNIDA (Zanobini et al., 2016) in all clinical centers in Italy.

Furthermore, the teleNIDA scores were compared to the ADOS-2 concern categories (convergent validity) and the ROC analyses were implemented to measure the strength of discrimination between toddlers with low-level vs high-level concerns. Our findings revealed that the teleNIDA is a promising and feasible remote approach to ASD screening. It demonstrated good discrimination between children who fell in the moderate‐to‐severe level of ADOS-2 concern vs. children who fell in either the little‐to‐no concern or the mild‐to‐moderate concern categories. The teleNIDA scoring is derived from the SORF rating form published by Dow et al. (2020) with some adjustments and adaptations to the duration and quantity of teleNIDA videos. Dow et al. (2020) examined the psychometric properties of the SORF as a screening tool in a sample of 228 toddlers (84 with ASD, 82 with developmental delay, and 62 typically developing children). The ROC analyses revealed that the SORF significantly discriminated between ADOS-2 concern classifications and the SORF total score showed specificity and sensitivity of 0.74.

Our data is consistent with these results and demonstrates good validity and agreement between the teleNIDA and the ADOS-2, the gold standard test for ASD diagnosis. In particular, the teleNIDA showed a specificity of 0.70 and a sensitivity of 0.83 at a cutoff of 15 for the total scores, a specificity of 0.67 and a sensitivity of 0.83 at a cutoff of 13 for social communication/interaction, and a specificity of 0.74 and a sensitivity of 0.75 at a cutoff of 2 for restricted/repetitive behaviors. The positive and negative predictive values for both total and subdomains were found to be adequate and similar to results obtained with other screening tools (Glascoe, 2005), such as SORF (Dow et al., 2020). The teleNIDA may offer a significant benefit for the diagnostic process, however, since PPVs are modest, additional clinical measures are recommended to decrease false positives and increase PPVs.

Based on these results, the teleNIDA may offer a new and more accessible option for remote screening of autism using a reliable system with a shorter timeframe (i.e., 5-min videos for a total duration of about 20 minutes) and different naturalistic contexts compared to other asynchronous tools. The SORF includes five videos for a total duration of a minimum of 30 minutes, but the research data is based on one-hour home observations and results with a shorter timeframe are not available (Dow et al., 2020). Moreover, the more recent BOSA is based on ADOS-2 coding, activities, and materials and does not consider observations of different everyday settings (Dow et al., 2021). The BOSA is very promising, but further empirical validation is still needed. Observing children in their naturalistic environment (home-setting) gives a view of the child during everyday activities that would not otherwise be accessible to clinicians, allowing for more family-centered recommendations.

The importance of telehealth methods for screening of ASD has increased substantially because of the COVID-19 pandemic. However, very few studies have reported validity data. A recent review by Stavropoulos et al. (2022) showed that only six studies published from 2010 to 2021 provided information on validity (sensitivity and/or specificity). Among these studies, only three used store-and-forward procedures and demonstrated good specificity and sensitivity values.

Notably, previous studies have been based mostly on American populations (Alfuraydan et al., 2020; Meimei & Zenghui, 2022). This is the first study that compared a telehealth screening tool with a conventional diagnostic assessment for ASD in a European country (i.e., Italy). Covering a wider geographical area allows us to examine the applicability of telehealth screening tools in different countries and to understand how family, environmental, and cultural characteristics influence telehealth approaches for ASD, which is extremely challenging.

By considering the item-level results, we found that most items showed moderate-to-high AUC values, which supports good discrimination values between children with low-level vs high-level of ASD risk. In particular, 13 of the 22 items showed significant discrimination between concern classifications with AUC values above 0.65. Interestingly, eight items obtained the best discrimination values (with an AUC > 0.75), all within the social communication and interaction domain. These results are similar to the previous study on SORF (Dow et al., 2020). We found an overlap with the six SORF items with the best psychometric properties (i.e., poor eye gaze directed to faces, limited showing and pointing, limited coordination of nonverbal communication, less interest in people than objects, repetitive use of objects, and excessive interest in particular objects/actions/activities). However, we did not find the best psychometric properties for items within the restricted/repetitive behaviors domain. It is possible that teleNIDA activities and guidelines provide more opportunities to detect communication, social-emotional reciprocity, and independent play skills and lack opportunities to explore repetitive, restricted, and stereotyped behavioral patterns (Stronach & Wetherby, 2014). Our results are consistent with previous studies on screening measures for ASD that demonstrated that children with later diagnosis of ASD have more difficulties in their social communication abilities; however, they did not show atypical sensory and repetitive behaviors (Dow et al., 2017; Rowberry et al., 2015). Different environmental setups for exploring these behaviors are needed to collect more informative and comprehensive materials.

Finally, this study examined the predictive validity of the teleNIDA on the diagnosis of ASD. The results maintained good validity parameters and discrimination properties in differentiating ASD from non-autistic groups at 36 months. The teleNIDA showed a specificity of 0.70 and a sensitivity of 0.76 at a cutoff of 17 for the total scores, a specificity of 0.70 and a sensitivity of 0.71 at a cutoff of 15 for social communication/interaction, and a specificity of 0.80 and a sensitivity of 0.71 at a cutoff of 2 for restricted/repetitive behaviors. In addition, this study provided optimal cutoffs that resulted in acceptable positive and negative predictive values for detecting autism. Overall, these preliminary data suggested that the teleNIDA may be helpful in support the clinical judgment in toddlers at risk for ASD facilitating the administration of a comprehensive diagnostic evaluation that will drive intervention recommendations.

### Limitations and Future Directions

Our study has some limitations that should be mentioned. First, it focused on a relatively small sample of toddlers; however, the assessment of this sample was restricted to a specific COVID-19 pandemic period. Further replications of independent and larger cohorts would help generalize our findings. Second, our sample is skewed towards at-risk toddlers seen for comprehensive ASD evaluations and did not include typically developing children. For this reason, our results (i.e., optimal cutoffs, positive and negative predictive values) should be used with caution and clinical judgment should always be considered. Third, the number of toddlers with atypical development outcomes at 36 months was small (n = 6), limiting our study to include them as a specific diagnostic group. Future research is needed to replicate these data in larger samples, including validity parameters in discriminating ASD and typical development groups versus atypical development group. Fourth, we did not develop the teleNIDA guidelines (instructions and recording duration) for parents/caregivers through a standardized procedure. Ad hoc written instructions accompanied by clear images were provided to guide the parents through the interaction and recording procedures, including activities to elicit child typical/atypical behaviors in different ASD-related domains. Prior to creating the teleNIDA coding, 14 clinicians from 7 different pediatric institutes selected and shared activities based on the scientific literature and their strong clinical expertise in ASD diagnosis and assessment. The main purpose of these instructions was to standardize recording settings and procedures for eliciting specific behaviors in different ASD-related domains. For these reasons, replication and validation data are needed to determine the accuracy of teleNIDA's instructions and recording durations.

Besides these limitations, the strengths of this study also need to be acknowledged. The current findings demonstrated good validity data of a novel and more feasible telehealth approach administered in home settings for remote level 2 screening of ASD during the COVID-19 pandemic, when enforced restrictions significantly affected the healthcare system. The teleNIDA offers the possibility to assess child behaviors remotely and in a naturalistic setting (without potential interference caused by the presence of clinicians), allowing caregivers to be more involved than in traditional, in-person evaluations. However, the disadvantages of telehealth tools should be noted, including the lack of information about cognitive skills that may reduce clinician’s confidence in the diagnosis and the lack of ability to interact directly with the child, which is an essential part of the assessment process. Furthermore, important barriers to conducting telehealth are associated with technological requirements, including lack of user-friendly technological devices, poor internet connections (lack of broadband coverage), and difficulties in uploading videos. These difficulties may affect the quality of observations and the adequacy of the child’s assessment.

Beyond the COVID-19 pandemic period, the use of remote tools for level 2 screening of ASD can offer a unique perspective on the child’s clinical profile and help clinicians in the referral process for a comprehensive diagnostic evaluation. Novel screening approaches with more feasible remote methods may speed up the diagnostic process and guide intervention recommendations to improve the quality of life of children with ASD and their families.

## Supplementary Information

Below is the link to the electronic supplementary material.

Electronic supplementary material 1 (DOCX 62 kb)
