# Supplementary material for: The teleNIDA: Early Screening of Autism Spectrum Disorder Through a Novel Telehealth Approach
Source: J Autism Dev Disord. 2023 Feb 23;54(5):1680–90. doi: 10.1007/s10803-023-05927-6 (PMC9946866; doi:10.1007/s10803-023-05927-6)
Supplement: Supplementary file 1 — Electronic supplementary material 1 (DOCX 62 kb) [file 10803_2023_5927_MOESM1_ESM.docx]

**teleNIDA guidelines for parents/caregivers**

For the interest of readers, we will provide a schematic and an *ad hoc* English translation of the original teleNIDA guidelines for parents.

**1. FREE-PLAY (5 minutes)**

**
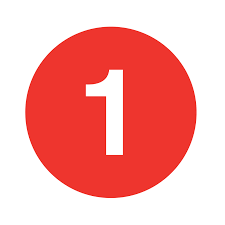
**(You can choose different toys, such as cars/trucks, balls, puzzles, baby dolls, stuffed animals, and blocks)

**
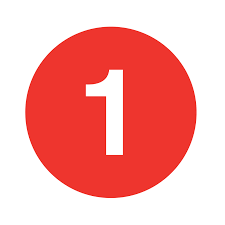
**
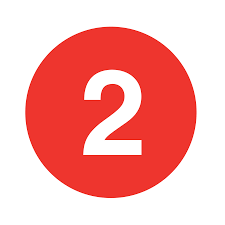
**PICK 2 OR 3 TOYS AND LET THE CHILD PLAY**


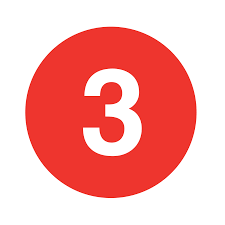
**SIT BACK WHILE HE/SHE IS PLAYING**

**
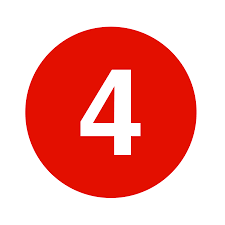
RESPOND NORMALLY TO YOUR CHILD IF SHE/HE INITIATES AN INTERACTION**

**AT THE END OF FREE-PLAY, CALL YOUR CHILD’S NAME ONCE WHILE HE/SHE IS NOT LOOKING AT YOU**


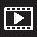


You should use a **normal speaking tone**

**If your child does not respond** to his/her name, try to **call him/her up to 3 times**


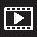


**2. PLAY WITH PARENTS/CAREGIVERS (5 minutes)** **
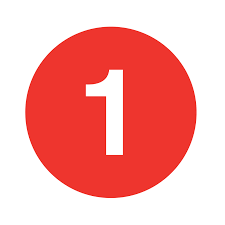
**

**BEFORE PLAYING, CALL YOUR CHILD’S NAME ONCE WHILE HE/SHE IS NOT LOOKING AT YOU**

**
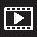
**

**If your child does not respond** to his/her name, try to **call him/her up to 3 times**


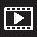


You should use a **normal speaking tone**


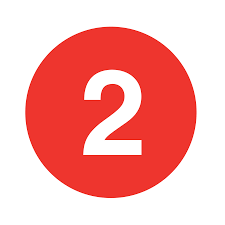


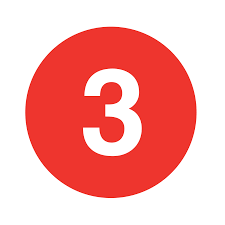
**PLAY WITH YOUR CHILD AS YOU USUALLY DO**

**DURING THE INTERACTION, CHOOSE A TOY IN THE ROOM, POINT AT IT, AND SAY “(Child’s name), LOOK!”**


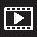


Point at the toy while the child is not looking at you

You should **indicate the toy without saying the name of the object** (i.e., “Bill, look!” and NOT “Bill, look at the ball! “)

You should use a **normal speaking tone**


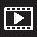


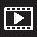


**
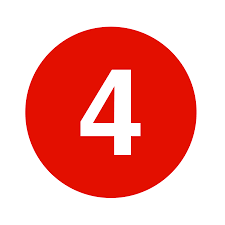
**

**DURING THE INTERACTION, INTERRUPT THE ACTIVITY THAT YOUR CHILD IS DOING**

**
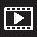
**

It is important that **the child is playing when you interrupt the activity**

You can interrupt the activity in many different ways, such as blocking toy play or moving the toy away from the child

**
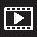
**

**
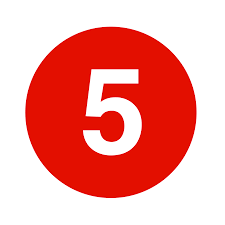
**

**IGNORE YOUR CHILD AND MOVE AWAY**

**
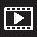
**

**Move away when your child is playing**

If your child approaches you, respond as you normally would

**
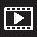
**

**
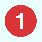
3. MEALTIME (5 minutes)**

**MAKE A VIDEO RECORDING DURING BREAKFAST/LUNCH/DINNER**

You and your child **should both be visible**


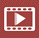


**
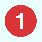
4. BOOK SHARING (5 minutes)**

**MAKE A VIDEO RECORDING WHILE YOU READ A BOOK WITH YOUR CHILD**

You and your child **should both be visible**


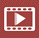


| **SOCIAL COMMUNICATION AND INTERACTION** | **ITEM DESCRIPTION** |
| --- | --- |
| 1. Limited sharing warm, joyful expressions | Minimal change in facial expressions |
| 2. Reduced facial expressions | Lack of a variety of facial expressions of affect |
| 3. Limited sharing interests | Lack of directing a person's attention to objects, actions or events of interest (e.g., initiating joint attention) |
| 4. Lack of response to name | Lack of response to name by looking at the person immediately after the child’s name is called |
| 5. Poor eye gaze directed to face | Lacks of eye gaze to caregiver during face-to-face interaction |
| 6. Limited showing and pointing | Limited use of showing (holds an object out toward another person without giving the object to reference another’s attention to the object) and pointing gestures (uses the index finger to reference another’s attention to an object, coordinated with eye gaze directed to a person immediately before, during, or after the point) |
| 7. Using another person’s hand as tool | Takes or moves another person's hand or body part as if it was a tool |
| 8. Limited directed consonant sounds | Limited consonant sounds as a communicative signal |
| 9. Limited coordination of nonverbal communication | Lack of coordination of at least three of the following behaviors simultaneously (gaze; facial expression; gesture; and sound) |
| 10. Less interest in people than objects | The child is less interested in people than objects when objects are available |
| 11. Limited reciprocal social play | Child does not show reciprocal, shared play actions with another person |
| **RESTRICTED AND REPETITIVE BEHAVIORS** |  |
| 12. Repetitive use of objects | The same movement repeated at least 3 consecutive times (e.g, 3 knocks down and rolls) |
| 13, Repetitive body movements | Stereotyped or repetitive movements or posturing of child’s body, arms, hands, or fingers |
| 14. Repetitive speech/intonation | Stereotyped speech that contains sounds that are produced at least 3 times in a row or close in time, or speech with unusual intonation. |
| 15. Ritualized patterns of behavior | Ritualistic patterns of behaviors (a sense of insistence on sameness, completeness, deliberateness, or precision) |
| 16. Marked distress over change | Intense distress when an object is removed |
| 17. Excessive interest in particular objects | Intense interests in particular objects |
| 18. Clutches particular objects | The child holds onto an object and does not release the object easily |
| 19. Sticky attention to objects | The child does not shift gaze away from an available object within 10 seconds |
| 20. Fixation on parts of objects | A narrow focus on or preoccupation with parts of objects |
| 21. Adverse response to sensory stimuli | Over-responsiveness to sensory input |
| 22. Unusual sensory exploration/interest | Unusual or prolonged sensory examination of objects |

**Item description of the teleNIDA** (adapted from SORF, Dow et al., 2020)

**Scoring teleNIDA** (adapted from SORF, Dow et al., 2020)

|  | **FREE-PLAY** | **PLAY WITH PARENT** | **MEALTIME** | **BOOK SHARING** | ***MEAN*** |
| --- | --- | --- | --- | --- | --- |
| **SOCIAL COMMUNICATION AND INTERACTION (1)** | | | | | |
| **1. Limited sharing warm, joyful expressions** | \|  \| \| --- \| | \|  \| \| --- \| | \|  \| \| --- \| |  | \|  \| \| --- \| |
| **2. Reduced facial expressions** |  | \|  \| \| --- \| | \|  \| \| --- \| | \|  \| \| --- \| | \|  \| \| --- \| |
| **3. Limited sharing interests** | \|  \| \| --- \| | \|  \| \| --- \| | \|  \| \| --- \| | \|  \| \| --- \| | \|  \| \| --- \| |
| **4. Lack of response to name** | \|  \| \| --- \| | \|  \| \| --- \| |  |  | \|  \| \| --- \| |
| **5. Poor eye gaze directed to face** | \|  \| \| --- \| | \|  \| \| --- \| | \|  \| \| --- \| |  | \|  \| \| --- \| |
| **6. Limited showing and pointing** | \|  \| \| --- \| | \|  \| \| --- \| |  |  | \|  \| \| --- \| |
| **7. Using another person’s hand as tool** |  | \|  \| \| --- \| |  | \|  \| \| --- \| | \|  \| \| --- \| |
| **8. Limited directed consonant sounds** | \|  \| \| --- \| | \|  \| \| --- \| | \|  \| \| --- \| | \|  \| \| --- \| | \|  \| \| --- \| |
| **9. Limited coordination of nonverbal communication** | \|  \| \| --- \| | \|  \| \| --- \| | \|  \| \| --- \| | \|  \| \| --- \| | \|  \| \| --- \| |
| **10. Less interest in people than objects** | \|  \| \| --- \| | \|  \| \| --- \| | \|  \| \| --- \| | \|  \| \| --- \| | \|  \| \| --- \| |
| **11. Limited reciprocal social play** |  | \|  \| \| --- \| |  |  | \|  \| \| --- \| |
| **TOTAL (1)** |  |  |  |  | \|  \| \| --- \| |
| **RESTRICTED AND REPETITIVE BEHAVIORS (2)** | | | | | |
| **12. Repetitive use of objects** | \|  \| \| --- \| | \|  \| \| --- \| | \|  \| \| --- \| | \|  \| \| --- \| | \|  \| \| --- \| |
| **13, Repetitive body movements** | \|  \| \| --- \| | \|  \| \| --- \| | \|  \| \| --- \| | \|  \| \| --- \| | \|  \| \| --- \| |
| **14. Repetitive speech/intonation** | \|  \| \| --- \| | \|  \| \| --- \| | \|  \| \| --- \| | \|  \| \| --- \| | \|  \| \| --- \| |
| **15. Ritualized patterns of behavior** | \|  \| \| --- \| | \|  \| \| --- \| | \|  \| \| --- \| | \|  \| \| --- \| | \|  \| \| --- \| |
| **16. Marked distress over change** | \|  \| \| --- \| | \|  \| \| --- \| | \|  \| \| --- \| | \|  \| \| --- \| | \|  \| \| --- \| |
| **17. Excessive interest in particular objects** | \|  \| \| --- \| | \|  \| \| --- \| | \|  \| \| --- \| | \|  \| \| --- \| | \|  \| \| --- \| |
| **18. Clutches particular objects** | \|  \| \| --- \| | \|  \| \| --- \| | \|  \| \| --- \| |  | \|  \| \| --- \| |
| **19. Sticky attention to objects** | \|  \| \| --- \| | \|  \| \| --- \| | \|  \| \| --- \| | \|  \| \| --- \| | \|  \| \| --- \| |
| **20. Fixation on parts of objects** | \|  \| \| --- \| | \|  \| \| --- \| | \|  \| \| --- \| | \|  \| \| --- \| | \|  \| \| --- \| |
| **21. Adverse response to sensory stimuli** | \|  \| \| --- \| | \|  \| \| --- \| | \|  \| \| --- \| | \|  \| \| --- \| | \|  \| \| --- \| |
| **22. Unusual sensory exploration/interest** | \|  \| \| --- \| | \|  \| \| --- \| | \|  \| \| --- \| | \|  \| \| --- \| | \|  \| \| --- \| |
| **TOTAL (2)** |  |  |  |  | \|  \| \| --- \| |
| **TOTAL (1) + (2)** |  |  |  |  | \|  \| \| --- \| |
